# Compensatory load redistribution in Labrador retrievers when carrying different weights – a non-randomized prospective trial

**DOI:** 10.1186/s12917-016-0715-7

**Published:** 2016-06-07

**Authors:** Barbara Bockstahler, Alexander Tichy, Patricia Aigner

**Affiliations:** Department for Small Animals and Horses, Small Animal Surgery, Section for Physical Therapy and Rehabilitation, University of Veterinary Medicine, Vienna, Austria; Department for Biomedical Sciences, Platform Bioinformatics and Biostatistics, University of Veterinary Medicine, Vienna, Austria

**Keywords:** Carrying weights, Dog, Ground reaction forces, Hunting

## Abstract

**Background:**

Retrievers are dogs particularly bred to retrieve birds or other small game, for the retrieval, the dogs are typically sent to the place where the shot game has fallen or to search the field for the wounded but still live game in order to return them to the hunter as quickly as possible. Examples of game animals are pheasants, mallard ducks and rabbits. For training, dummies with a variety of weights are used to simulate the retrieval of various types of game. The aim of this non-randomized prospective study was to investigate if peak vertical force, vertical impulse and paw pressure contact area are increased in the forelimbs when carrying different weights, and if the symmetrical weight distribution between contralateral limb pairs is disturbed. Ten actively working Labrador retrievers were walked over a pressure plate with or without carrying 0.5, 2.0 and 4.0 kg dummies. The aim of this study was to determine if vertical ground reaction forces and paw pressure contact area are increased in the forelimbs when carrying different weights, and if symmetrical weight distribution is disturbed between contralateral limb pairs.

**Results:**

Peak vertical force and vertical impulse were significantly increased in the forelimbs and decreased in the hindlimbs in all weight carrying conditions.

**Conclusions:**

These results demonstrate the significant effects of carrying weight in the mouth on the ground reaction forces, which likely produce additional stress on the forelimb joints. Carry of game or a dummy is likely to alter the forelimb load distribution.

## Background

**Fig. 1:**
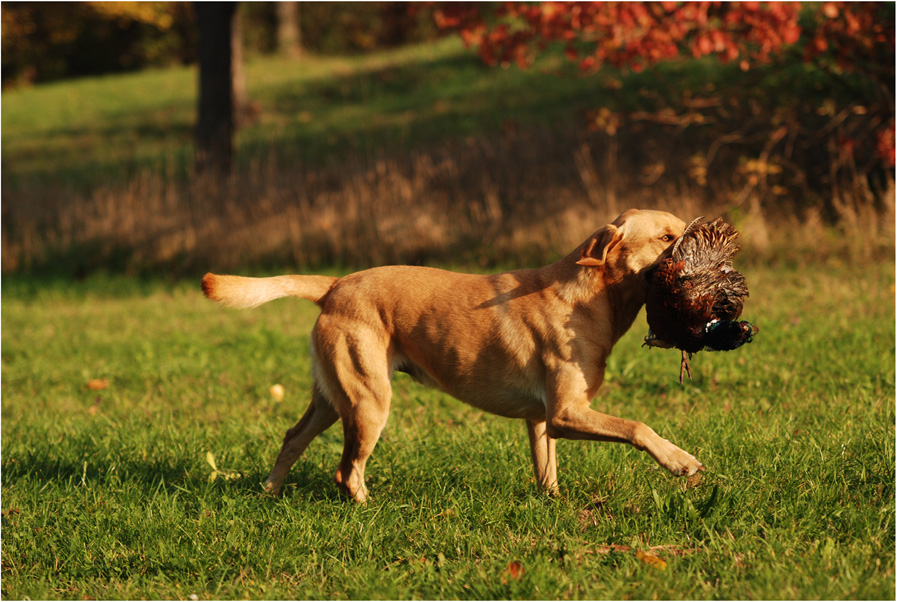
A Labrador retriever retrieving a pheasant (with permission of Elli Winter, www.moorhunde.de, www.work-labs.at)

**Fig. 2:**
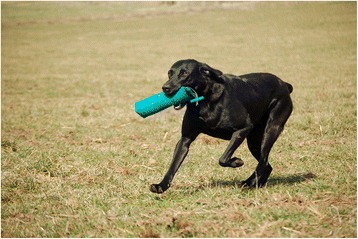
A Labrador retriever retrieving a standard dummy (with permission of Elli Winter, www.moorhunde.de, www.work-labs.at)

Retrievers are dogs particularly bred to retrieve birds or other small, hunted game. For the retrieval, the dogs are typically sent to the place where the shot game has fallen or to search the field for the wounded but still live game in order to return them to the hunter as quickly as possible. Examples of game animals are pheasants (Fig. 1), with a weight between 1.0 and 1.5 kg, mallard ducks weighing between 0.7 and 1.4 kg, and rabbits, with a body mass of 2–6 kg. For training, dummies with a variety of weights, such as 0.5, 1, 2 or 4 kg, are used to simulate the retrieval of various types of game. Additionally, the dogs are frequently entered in hunting tests (working tests) where a standard 0.5 kg dummy is retrieved rather than a game animal (Fig. 2). These tests have gained in popularity with individuals who wish to respect the dogs’ natural dispositions in a way that does not involve hunting, as, the number of starts in Austria increased from 600 in the 2004 season to 1200 starts in 2013 [1]. With the upsurge in sporting/working activities comes the increasing importance of veterinary sports medicine, and additional focus has been placed on related special injury risks. For example, it was shown that 32 % of agility dogs incur at least one injury [2]. A survey of gundog lameness and injuries in Great Britain showed that the incidence of injuries/lameness in two shooting seasons was 25 %, including fractures, muscular injuries and articular pathologies [3].

Sports medicine veterinarians should have an in-depth knowledge of exercise physiology and biomechanics during sport and work. Research has shown that flexion of the shoulder joint increases with the height of the fence during the takeoff and bascule phases of a jump [4], and that extremely high (4.5× the bodyweight) peak vertical force (PFz) occurs in the forelimbs when landing from a hurdle jump at high speed [5]. With respect to retrieval dogs, carry of game or a dummy is likely to alter the forelimb load distribution. The aim of this non-randomized prospective study was to determine if PFz, vertical impulse (IFz) and paw pressure contact area (PCA) are increased in the forelimbs when carrying different weights, if step length (SL) and stand phase duration (SPD) changes and if symmetrical weight distribution is disturbed between contralateral limb pairs.

## Results

### Ground reaction forces

The calculated PFz, IFz and the symmetry index (SI) for each condition are shown in Table 1. The mixed model revealed significant effects for both PFz and IFz for the measurement condition (*P* = 0.00) and the front versus hind quarter (*P* = 0.00) but not for the contralateral body side (*P* = 0.46). The PFz and IFz significantly increased in forelimbs and decreased in hindlimbs with increasing dummy weight (*P*-values are given in Table 1). The mixed model revealed significant effects for SIPFz for the the front versus hind quarter (lower in front legs, *P* = 0.001), but not for the measurement condition, no significant effects were found for SIIFz.

**Table 1:** Peak vertical forces and vertical impulses. Values are presented as mean ± SD % total force (%TF) and the symmetry index (SI) during each of the four measurement conditions

	LF (%TF)	RF (%TF)	SI (%)	LH (%TF)	RH (%TF)	SI (%)
Peak vertical force						
W1	32.1 ± 1.4*	32.1 ± 1.4*	2.0 ± 1.5	17.8 ± 1.5*	17.9 ± 1.2*	3.1 ± 2.1
W2	33.0 ± 1.6*	33.2 ± 1.5*	1.8 ± 1.8	16.8 ± 1.6*	17.0 ± 1.5*	3.5 ± 1.9
W3	34.7 ± 1.8*	35.0 ± 1.7*	2.1 ± 1.5	15.0 ± 1.7*	15.2 ± 1.8*	3.3 ± 2.5
W4	37.3 ± 1.6*	37.7 ± 1.3*	1.8 ± 1.5	12.4 ± 1.5*	12.6 ± 1.5*	5.1 ± 3.1
Vertical impulse						
W1	32.5 ± 1.4*	32.1 ± 0.9*	3.4 ± 3.0	17.8 ± 1.1*	17.7 ± 0.8*	2.5 ± 2.2
W2	33.4 ± 1.2^+^	33.7 ± 0.9*	3.4 ± 2.3	16.3 ± 1.0*	16.6 ± 0.8*	3.4 ± 2.5
W3	35.4 ± 1.3*	35.6 ± 0.9*	2.6 ± 2.0	14.5 ± 1.1*	14.5 ± 0.8*	2.8 ± 2.2
W4	38.2 ± 1.7*	38.2 ± 1.3*	4.5 ± 3.2	11.7 ± 1.1*	11.9 ± 1.1*	3.3 ± 2.9

W1 = unimpeded walking without a weight; W2 = carrying a standard 0.5 kg dummy; W3 = carrying a 2.0 kg dummy; W4 = carrying a 4.0 kg dummy

*LF* left forelimb, *RF* right forelimb, *LH* left hindlimb, *RH* right hindlimb

*P ≤ 0.02 *vs* other conditions; ^+^ *P* = 0.02 *vs* W1

### Pressure contact area

The mixed model revealed no significant effects for the measurement condition but for the the front versus hind quarter (front larger compared to hind limbs, *P* = 0.00, Table 2).

**Table 2:** Pressure contact area during the four measurement conditions

Paw contact area (cm^2^)				
	LF	RF	LH	RH
W1	46.4 ± 4.8	45.6 ± 4.2	36.7 ± 3.6	37.2 ± 2.9
W2	46.4 ± 4.7	45.9 ± 4.0	35.8 ± 3.5	35.9 ± 3.4
W3	47.7 ± 4.5	47.3 ± 3.9	35.2 ± 3.5	35.4 ± 3.4
W4	48.7 ± 4.3	48.4 ± 3.9	32.7 ± 4.5	33.1 ± 3.9

Values are presented as mean ± SD. W1 = unimpeded walking without a weight; W2 = carrying a standard 0.5 kg dummy; W3 = carrying a 2.0 kg dummy; W4 = carrying a 4.0 kg dummy

*LF* left forelimb, *RF* right forelimb, *LH* left hindlimb, *RH* right hindlimb

### Step length (Table 3)

**Table 3:** Step length (m) and stand phase duration (s). Values are presented as mean ± SD

	LF	RF	LH	RH
Step length (m)				
W1	0.78 ± 0.0*	0.78 ± 0.1*	0.78 ± 0.0**	0.78 ± 0.1
W2	0.75 ± 0.1	0.76 ± 0.1	0.76 ± 0.1	0.75 ± 0.1
W3	0.75 ± 0.1	0.75 ± 0.1	0.75 ± 0.1	0.75 ± 0.1
W4	0.72 ± 0.0*	0.72 ± 0.1*	0.71 ± 0.0*	0.72 ± 0.0*
Stand phase duration (s)				
W1	0.43 ± 0.1	0.43 ± 0.1*	0.41 ± 0.0	0.41 ± 0.0
W2	0.47 ± 0.1	0.47 ± 0.1	0.43 ± 0.0	0.44 ± 0.1
W3	0.46 ± 0.1	0.46 ± 0.1	0.42 ± 0.1	0.42 ± 0.1
W4	0.48 ± 0.0	0.48 ± 0.0	0.41 ± 0.0	0.42 ± 0.0

W1 = unimpeded walking without a weight; W2 = carrying a standard 0.5 kg dummy; W3 = carrying a 2.0 kg dummy; W4 = carrying a 4.0 kg dummy

*LF* left forelimb, *RF* right forelimb, *LH* left hindlimb, *RH* right hindlimb

*P ≤ 0.02 *vs* all other conditions

**P = 0.00 *vs* W3 and W4

The mixed model revealed a significant effects for the measurement condition (*P* = 0.00) but not for the front versus hind quarter and the contralateral body side.

SL was significantly longer in forelimbs during W1 compared to all other conditions, but did not differ between W2 and W3. In the hind limbs SL was significant shorter while carrying the 4 kg dummy compared to all other conditions; no significant differences were found between W1, W2 and W3 in the right hindlimb, whereas in the left hind limb SL during W1 was longer compared to W3 (*P*-values are given in Table 3).

### Stand phase duration (Table 3)

The mixed model revealed a significant effect for the measurement condition (*P* = 0.02), the ANOVA detected this difference significant for the right forelimb (shorter during W1 compared to all other conditions (*P*-values are given in Table 3). Further the mixed model revealed a significant effect for the front versus hind quarter (front limbs longer SPD, *P* = 0.00) but not for the contralateral body side.

## Discussion

The results of this study support the hypothesis that the carrying of dummies alters the ground reaction force distribution but not the paw contact area. When the dogs were carrying dummies, PFz and IFz increased in the forelimbs and decreased in the hindlimbs, with no change in contralateral limb symmetry. Notably, the SI of the hindlimbs revealed during W4 conditions was slightly higher than described in normal, non-lame dogs [6–8]. These results suggest that the weight of the dummy is transferred to forelimbs, which could lead to additional stress on corresponding joints, muscles and connective tissues.

It is known that weight bearing is accomplished via the humeral condyle [9] and that the load is then transferred to the antebrachium via the radial head and the trochlear notch of the ulna. It was shown that with applied load of 50, 100 and 150 N a significant difference exists in the force distribution between the proximal articular surfaces of the radius and ulna, for example if a 100 N load was applied, the measured radial force was 125. ± 26.3 N and 119.0 ± 29.15 N, but the ratio of the mean force remained close to a 50:50 distribution regardless of the applied load [10]. Supraphysiological cartilage pressures can damage the cartilage matrix and subchondral bone [10] and contribute to the pathogenesis of osteoarthritis [11]. Thus, only dogs with sound elbow joints should be used for retrieving, and they should be checked regularly by veterinarians for potential orthopaedic problems. Furthermore, excessive stress could lead to micro-damage or fracture of the subchondral trabecular bone, and thus play a role in the pathogenesis of fragmented medial coronoid process [12]. Although there are no currently available studies investigating the relationship between retrieving work and elbow dysplasia and/or osteoarthritis, veterinarians should advise owners and trainers to carefully train young hunting dogs to ensure that the retrieval weights do not overstress the musculoskeletal system. Even this study was performed with working retrievers, the results might be applied also to other sporting dogs (like obedience dogs) and any other dog carrying weights while playing.

The results of this study also demonstrate load redistribution, thus involving the muscles of the back and cervical spine. Further studies using electromyographic techniques should be performed to investigate this in detail.

One limitation of the present study is that the measurements were taken in a laboratory environment, and thus may not accurately reflect the motions performed by dogs in the field. Moreover, all measurements were taken while the animals were walking, whereas game retrieval typically occurs quickly, often at a gallop. The peak vertical forces increase with velocity, while vertical impulses decrease, due to the shorter stance phase [13, 14]. One study reported PFz values of 2.2× the bodyweight in the trailing forelimb and 1.6× the bodyweight in the leading hindlimb of galloping dogs [15], suggesting that the force redistribution described in the present study is likely to be more pronounced in dogs moving at a gallop. Although this study only included Labrador retrievers, the results are not influenced by breed differences, as has been reported previously [16, 17]. Furthermore, the walking velocity between animals and within trials was consistent, and it was shown that variance of PFz and IFz is low even in ranges between 1.5 and 2.2 m/s [18]. Finally, the pressure plates only register vertical forces, and thus the effects of carrying a weight on mediolateral and craniocaudal forces cannot be evaluated.

## Conclusions

The findings presented here indicate that carrying dummies of varying weight in the mouth impacts ground reaction forces in retrieving dogs. This is of scientific and clinical interest as it indicates that additional stress is exerted on the forelimb joints. Therefore, the training of developing dogs should be performed with special attention to the immature skeletal system, and dogs carrying weights on a regular basis should undergo routine orthopaedic examinations.

## Methods

The study was discussed and approved by the institutional ethics committee in accordance with Good Scientific Practice guidelines and national legislation (18/03/97/2014). Consent was obtained from the owners.

### Study design

The study was a non-randomized prospective trial. Sample size calculation was based on previous published results of vertical force measurements. The reported baseline PFz value for the forelimbs during unimpeded level walking is 31.46 ± 1.56 of total force (%TF) for the left forelimb and 31.29 ± 1.86 %TF for the right forelimb [19]. Thus, a total of ten Labrador retrievers was deemed sufficient to detect statistically significant differences with a one-tailed α of 0.05 at a power of 0.80 based on a cutoff value of 5 % for discrimination of PFz between unimpeded walking and weight-carrying conditions. Data were collected at the University of Veterinary Medicine between July and August 2014.

### Dogs

Ten active, privately owned, working (not retired) Labrador retrievers were included in the study. Dogs were included only if a thorough orthopaedic examination did not reveal any clinically detectable lameness or neurological impairment and the animals had a working test class of “novice” or higher. The dogs (6 female, 4 male) were 5.2 ± 2.4 years of age (range: 2–9 years) with a mean body mass of 27.2 ± 3.4 kg (range: 23–34 kg).

### Equipment and measurement procedure

**Fig. 3:**
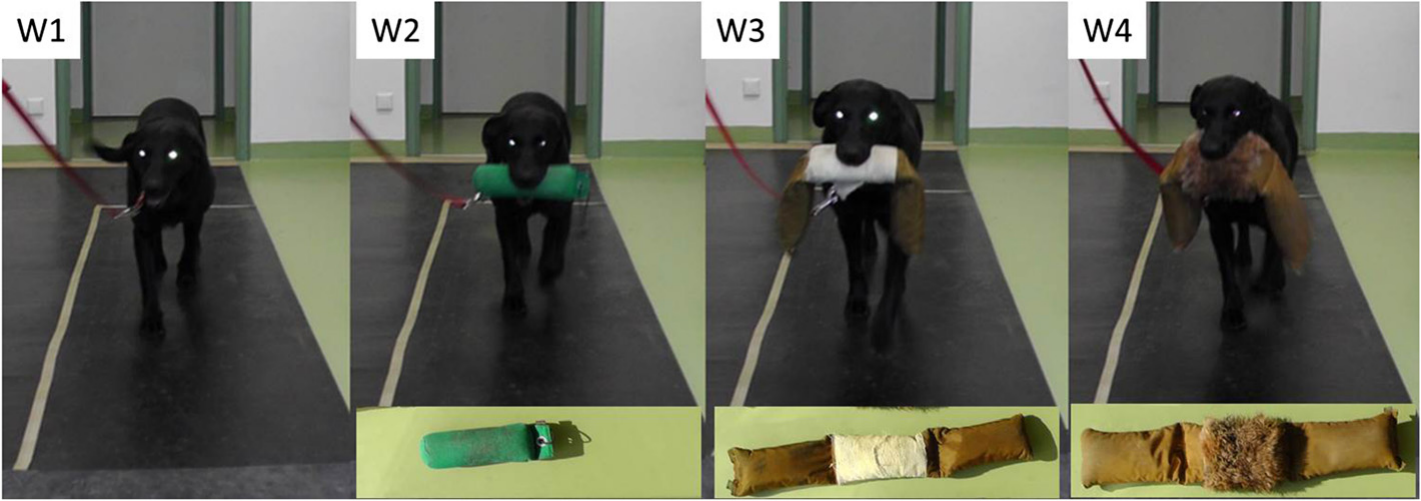
One of the dogs on the pressure plate. W1 = unimpeded walking without a weight; W2 = carrying a standard 0.5 kg dummy; W3 = carrying a 2.0 kg dummy; W4 = carrying a 4.0 kg dummy

All measurements were performed at the University of Veterinary Medicine Vienna. The pressure plate (FDM Type 2; Zebris Medical GmbH, Allgäu, Germany) had a measurement area of 203.2 × 54.2 cm with 15,360 sensors and a sampling rate of 100 H. The plate was mounted in the middle of a 7-m runway and covered with a rubber mat (2 mm thickness) to hide the measurement area and prevent slipping. The dogs were given sufficient time to acclimate to the testing facility and become accustomed to the equipment by walking freely in the room and over the plate. They were subsequently walked over the plate on a leash several times until they showed a smooth and harmonious gait pattern. This procedure was repeated before each of the measurement conditions. The dogs were walked at their own comfortable speed over the platform by their owners under four different conditions: W1 = unimpeded walking without a weight; W2 = carrying a 0.5 kg dummy; W3 = carrying a 2.0 kg dummy; W4 = carrying a 4.0 kg dummy (Fig. 3). The dogs were principally trained to carry the dummy in the middle and measurements were only taken if the dummy was carried correctly. Each dog performed the tests in the same order with a 5-min rest period in between: W1 followed by W2, W3 and W4. The dogs were walked several times over the plate for each condition to obtain a sufficient number (*n* = 5) of valid trials [19]. A trial was rated as valid if the dog walked in a straight line over the plate with the head in a straight-forward position without apparent change of velocity. The velocity of the dogs was calculated for the left forelimb based on the time between successive forelimb ground contacts, and trials were only accepted if the difference in velocity between W1 and the trials with the dummies was ≤ 0.3 m/s [13, 14]; the mean velocity of the dogs was 1.1 ± 0.1 m/s over all conditions.

### Data analysis

Pressure prints of the footfalls were manually identified from video recordings and matched with the corresponding limbs. Data were processed using specially developed software (Pressure Analyzer 1.3.0.2, Michael Schwanda). For each limb, the mean PFz, IFz, PCA (cm^2^), step length (SL (m)), and stance phase duration (SPD (s)) were calculated for each condition. To assess the compensatory effects of carrying weights, the force data given in Newton were normalized to the sum of all forces exerted by the four limbs and expressed as percentage of total force (%TF). To describe the symmetry between limb pairs, a symmetry index (SI) was calculated as a percent using the formula: SI (%) = abs (1-F_l_/F_r_)*100; where F = the ground reaction force parameter (PFz or IFz), _l_ = left fore- or hindlimb, _r_ = right fore- or hindlimb.

### Statistics

After testing for a normal distribution with a Kolmogorov–Smirnov test, data were analysed using mixed model analysis where measurement conditions, direction (front vs hind limbs) and side (left vs right) were included into the model. Differences between measurement conditions were analysed using Sidak’s post hoc procedure. In addition differences between measurement conditions for each leg were investigated using ANOVA for repeated measurements with Bonferroni’s alpha correction procedure. A *P* < 0.05 was considered statistically significant.

## Abbreviations

IFz, vertical impulse; PCA, pressure contact area; PFz, peak vertical force; SI, symmetry index; W1, unimpeded walking; W2, carrying a 0.5 kg dummy; W3, carrying a 2 kg dummy; W4, carrying a 4 kg dummy
